# Supplementary material for: Modeling full-scale leaf venation networks
Source: PLoS Comput Biol. 2025 Jul 21;21(7):e1013292. doi: 10.1371/journal.pcbi.1013292 (PMC12303385; doi:10.1371/journal.pcbi.1013292)
Supplement: S1 Text — (PDF) [file pcbi.1013292.s001.pdf]

Supplemental Information

Lars Erik J. Skjegstad & Julius B. Kirkegaard

June 2, 2025

1 Effects of perturbing the segmentation

The classification of the pixels in the extraction process uses a threshold value set to $p = 0.5$. Variations of this parameter can naturally change the output network, where a larger value of p results in a smaller network, as fewer pixels are classified as veins. To understand the influence of variations to the preprocessing pipeline, we consider a sample of *S. albus*, and run the same experiment as shown in Fig. 4a–b, (main text), for $p \in [0.1; 0.9]$. Fig. S1 shows the outcome of this experiment. We observe that the value of the optimal sink fluctuation σ^* only weakly depends on the preprocessing parameter for reasonable variations of p, i.e. the determined value of σ^* is well-defined for a broad range ($0.3 \leq p \leq 0.7$).

2 Faster computation of $\boldsymbol{A}^\dagger$

We consider the equation

$$\boldsymbol{p} = \boldsymbol{A}^\dagger \boldsymbol{s}, \tag{S1}$$

where $\boldsymbol{p}$ and $\boldsymbol{s}$ are vectors of dimension n_n, $\boldsymbol{A}$ is an $n_\mathrm{n} \times n_\mathrm{n}$ weighted graph Laplacian matrix as defined in the main text, and † denotes the Moore-Penrose pseudo-inverse. The calculation of the pseudo-inverse is expensive, but we here show one approach leading to an expression where only a normal inverse needs to be computed.

Define the $(n_\mathrm{n} \times n_\mathrm{n} - 1)$ matrix

$$\boldsymbol{T} \equiv \begin{bmatrix} 1 & 1 & \dots & 1 & 1 \\ -1 & 0 & \dots & 0 & 0 \\ 0 & -1 & \dots & 0 & 0 \\ \vdots & \vdots & \ddots & \vdots & \vdots \\ 0 & 0 & \dots & -1 & 0 \\ 0 & 0 & \dots & 0 & -1 \end{bmatrix}. \tag{S2}$$

By construction, $\boldsymbol{T}$ has full column rank, and the same column space as $\boldsymbol{A}$. Since $\boldsymbol{A}$ and $\boldsymbol{A}^\dagger$ are symmetric, we have equality between the column spaces

$$C(\boldsymbol{T}) = C(\boldsymbol{A}) = C(\boldsymbol{A}^T) = C(\boldsymbol{A}^\dagger). \tag{S3}$$

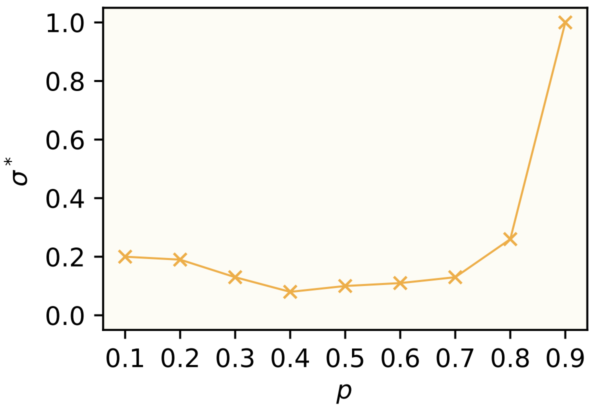

Figure S1: The optimal sink fluctuation σ^* as a function of the label probability p in network extraction from segmented image probabilities.

Furthermore, $\boldsymbol{T}$ has linearly independent columns (but not linearly independent rows), which implies that $\boldsymbol{T}^\dagger$ is a *left* inverse for $\boldsymbol{T}$, i.e.,

$$\boldsymbol{T}^\dagger \boldsymbol{T} = \boldsymbol{I}, \tag{S4}$$

and that $\boldsymbol{T}\boldsymbol{T}^\dagger \neq \boldsymbol{I}$ is a projection matrix onto $C(\boldsymbol{T}) = C(\boldsymbol{A}^\dagger)$. This implies that $\boldsymbol{T}\boldsymbol{T}^\dagger = \boldsymbol{A}\boldsymbol{A}^\dagger = \boldsymbol{A}^\dagger\boldsymbol{A}$, and that

$$\boldsymbol{A}^\dagger = \boldsymbol{T}\boldsymbol{T}^\dagger \boldsymbol{A}^\dagger. \tag{S5}$$

By definition, $\boldsymbol{A}^\dagger = \boldsymbol{A}^\dagger\boldsymbol{A}\boldsymbol{A}^\dagger$, so we can expand Eq. (S5) by writing

$$\begin{aligned}\boldsymbol{A}^\dagger &= \boldsymbol{T}\boldsymbol{T}^\dagger\boldsymbol{A}^\dagger\boldsymbol{A}\boldsymbol{A}^\dagger \\ &= \boldsymbol{T}\boldsymbol{T}^\dagger\boldsymbol{A}^\dagger\boldsymbol{T}\boldsymbol{T}^\dagger \\ &= \boldsymbol{T}\boldsymbol{T}^\dagger\boldsymbol{A}^\dagger(\boldsymbol{T}\boldsymbol{T}^\dagger)^T \\ &= \boldsymbol{T}\boldsymbol{T}^\dagger\boldsymbol{A}^\dagger(\boldsymbol{T}^T)^\dagger\boldsymbol{T}^T,\end{aligned} \tag{S6}$$

where we have used the fact that projection matrices are symmetric, as well as $(\boldsymbol{T}\boldsymbol{T}^\dagger)^T = (\boldsymbol{T}^T)^\dagger\boldsymbol{T}^T$, which is a basic property of the Moore-Penrose pseudo-inverse.

The goal is now to show that $\boldsymbol{T}^\dagger\boldsymbol{A}^\dagger(\boldsymbol{T}^T)^\dagger = (\boldsymbol{T}^T\boldsymbol{A}\boldsymbol{T})^{-1}$. Combined with Eq. (S6) this will give us an expression for $\boldsymbol{A}^\dagger$ which can be computed without any pseudo-inverse matrices, which is where we get the significant speed-up. We

use the following identity:

$$\begin{aligned}\boldsymbol{I} &= \boldsymbol{T}^\dagger \boldsymbol{T} \\ &= \boldsymbol{T}^\dagger \boldsymbol{T}\boldsymbol{T}^\dagger \boldsymbol{T} \\ &= \boldsymbol{T}^\dagger \boldsymbol{A}^\dagger \boldsymbol{A}\boldsymbol{T} \\ &= \boldsymbol{T}^\dagger \boldsymbol{A}^\dagger \boldsymbol{A}\boldsymbol{A}^\dagger \boldsymbol{A}\boldsymbol{T} \\ &= \boldsymbol{T}^\dagger \boldsymbol{A}^\dagger (\boldsymbol{T}\boldsymbol{T}^\dagger)^T \boldsymbol{A}\boldsymbol{T} \\ &= \boldsymbol{T}^\dagger \boldsymbol{A}^\dagger (\boldsymbol{T}^\dagger)^T \boldsymbol{T}^T \boldsymbol{A}\boldsymbol{T},\end{aligned} \tag{S7}$$

which implies that $\boldsymbol{T}^\dagger \boldsymbol{A}^\dagger (\boldsymbol{T}^\dagger)^T$ is a left inverse for $\boldsymbol{T}^T \boldsymbol{A}\boldsymbol{T}$. Continuing from Eq. (S7), we have:

$$\begin{aligned}\boldsymbol{I} &= \boldsymbol{T}^\dagger \boldsymbol{A}^\dagger (\boldsymbol{T}^\dagger)^T \boldsymbol{T}^T \boldsymbol{A}\boldsymbol{T} \\ &= (\boldsymbol{T}^\dagger \boldsymbol{A}^\dagger (\boldsymbol{T}^\dagger)^T \boldsymbol{T}^T \boldsymbol{A}\boldsymbol{T})^T \\ &= (\boldsymbol{T}^T \boldsymbol{A}\boldsymbol{T})^T [\boldsymbol{T}^\dagger \boldsymbol{A}^\dagger (\boldsymbol{T}^\dagger)^T]^T \\ &= \boldsymbol{T}^T \boldsymbol{A}\boldsymbol{T}\boldsymbol{T}^\dagger (\boldsymbol{A}^\dagger)^T (\boldsymbol{T}^\dagger)^T \\ &= \boldsymbol{T}^T \boldsymbol{A}\boldsymbol{T}\boldsymbol{T}^\dagger \boldsymbol{A}^\dagger (\boldsymbol{T}^\dagger)^T.\end{aligned} \tag{S8}$$

This implies that $\boldsymbol{T}^\dagger \boldsymbol{A}^\dagger (\boldsymbol{T}^\dagger)^T$ is also a right inverse for $\boldsymbol{T}^T \boldsymbol{A}\boldsymbol{T}$. Thus,

$$\boldsymbol{T}^\dagger \boldsymbol{A}^\dagger (\boldsymbol{T}^\dagger)^T = (\boldsymbol{T}^T \boldsymbol{A}\boldsymbol{T})^{-1}. \tag{S9}$$

Inserting this into Eq. S6 finally gives us:

$$\begin{aligned}\boldsymbol{A}^\dagger &= \boldsymbol{T}\boldsymbol{T}^\dagger \boldsymbol{A}^\dagger (\boldsymbol{T}^T)^\dagger \boldsymbol{T}^T \\ &= \boldsymbol{T}(\boldsymbol{T}^T \boldsymbol{A}\boldsymbol{T})^{-1} \boldsymbol{T}^T,\end{aligned} \tag{S10}$$

which we use explicitly in our calculations.

3 Flow bi-directionality

For each non-zero output model edge width i, we define the bi-directionality β_i as

$$\beta_i \equiv 1 - \left| \frac{1}{n_\mathrm{n} - 1} \sum_k \mathrm{sgn}(f_i(\boldsymbol{s}(k))) \right|, \tag{S11}$$

i.e., $\beta \to 0$ implies that the edge is unidirectional (the flow always goes in the same direction), and $\beta \to 1$ implies that the edge is bi-directional (the flow is equally likely to go in either direction). This allows us to visualize the bi-directionality as a function of the output model edge width. Figs. S2a-c show this for different values of sink fluctuation σ for the same sample of *S. albus* as in Fig. 5b (main text) for zero, optimal, and full sink fluctuation σ, respectively. Here we see that with no sink fluctuation (Fig. S2a), all veins are unidirectional.

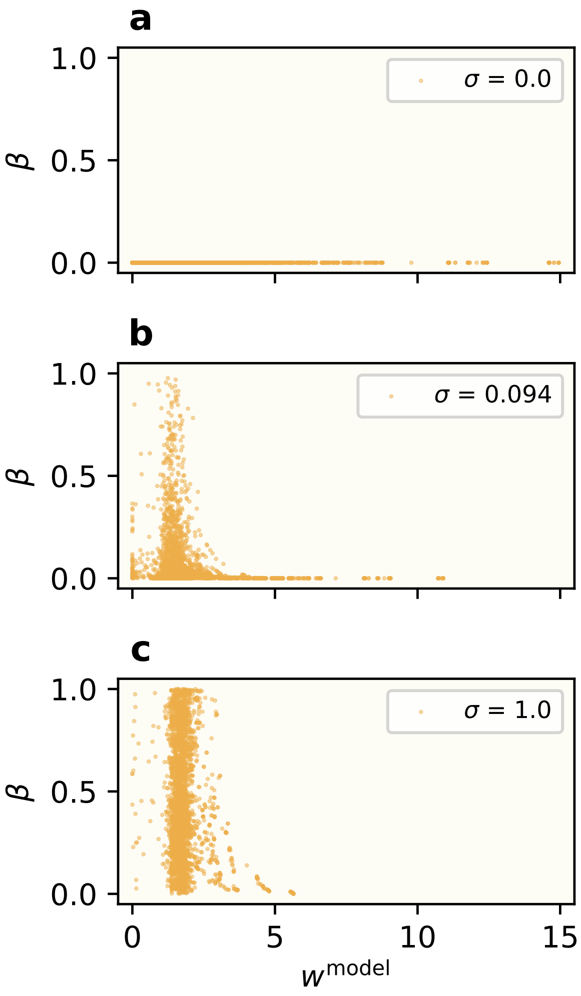

Figure S2: Bi-directionality β for the same sample of *S. albus* as in Fig. 5b (main text), shown for **(a)** Zero, **(b)** optimal, and **(c)** full sink fluctuation σ.

This is expected, as the network has a tree topology, and fluid can only flow one way in all veins. For full sink fluctuation (Fig. S2c), we see the opposite, namely that the bi-directionality is uniformly distributed, except for over the largest veins. For the optimal value of $\sigma = 0.094$ (Fig. S2b), we see a clearer difference between veins of lower and higher ranks. Larger veins, i.e., $w > 4$, are essentially unidirectional, whereas there is a markedly higher variance for smaller veins. Lastly we notice that the range of output widths decreases as sink fluctuation increases, as expected, due to the flattening of the hierarchy of vein widths.

Figure Legends

Figure S1 The optimal sink fluctuation σ^* as a function of the label probability p in network extraction from segmented image probabilities.

Figure S2 Bi-directionality β for the same sample of *S. albus* as in Fig. 5b (main text), shown for **(a)** Zero, **(b)** optimal, and **(c)** full sink fluctuation σ.
